# Comparison of Publication Rates for Musculoskeletal Oncology Abstracts Presented at National Meetings

**DOI:** 10.1155/2021/8326318

**Published:** 2021-03-05

**Authors:** Christopher D. Collier, Humzah A. Quereshy, Patrick J. Getty

**Affiliations:** ^1^Department of Orthopaedic Surgery, Indiana University School of Medicine, 1130 W Michigan Street, Fesler Hall 115, Indianapolis, IN 46202, USA; ^2^Department of Orthopaedics, University Hospitals Cleveland Medical Center, Case Western Reserve University, 11100 Euclid Avenue, Cleveland, OH 44106, USA

## Abstract

**Background:**

Scientific meetings provide a forum to disseminate new research and advance patient care. The American Academy of Orthopaedic Surgeons (AAOS), Connective Tissue Oncology Society (CTOS), and Musculoskeletal Tumor Society (MSTS) annual meetings are examples of such gatherings in the field of musculoskeletal oncology. After a review of select MSTS abstracts from 1991 to 1999 revealed a 41% publication rate in scientific journals, previous authors cautioned meeting attendees that the majority of abstracts may not survive rigorous peer review and may not be scientifically valid. Since two decades have passed, this study reexamined publication rates and characteristics in a contemporary and expanded cohort of oncology abstracts presented at the AAOS, CTOS, and MSTS annual meetings.

**Methods:**

1408 podium and poster abstracts from the AAOS (oncology-focused from 2013 to 2015), CTOS (2012 to 2014), and MSTS (2012 to 2014) annual meetings were reviewed to allow for a four-year publication window. Searches were performed with PubMed and Google Scholar databases to identify full-text publications using abstract keywords. Characteristics of each abstract and resulting publication were collected. Statistical analysis was performed using the chi-square and Kruskal-Wallis tests for time-independent comparisons, and the log-rank test after reverse Kaplan-Meier analysis for time-dependent comparisons.

**Results:**

Abstract publication rates overall were higher for podium presentations (67%, 280 of 415) compared to poster presentations (53%, 530 of 993; *p* < 0.001). When both abstract types were combined, differences between meetings did not meet statistical significance (AAOS: 65%, 106 of 162; CTOS: 57%, 521 of 909; MSTS: 54%, 183 of 337, *p*=0.06). Abstracts from AAOS meetings were more often published prior to the first day of the meeting (AAOS: 24%, 25 of 106; CTOS: 10%, 52 of 521; MSTS: 14%, 25 of 183; *p* < 0.01). After excluding previously published abstracts, AAOS abstracts had the shortest time to publication (median: 10.8 months, interquartile range (IQR): 4.4 to 18.8 months), compared to those from CTOS (16.0 months, 8.4 to 25.9 months, *p* < 0.01) and MSTS (15 months, 7.9 to 25.0 months, *p* < 0.01) meetings. CTOS abstracts were published in higher impact journals (median: 3.7, IQR: 2.9 to 5.9), compared to those from AAOS (2.9, 1.9 to 3.2, *p* < 0.01) and MSTS (3.1, 2.3 to 3.1, *p* < 0.01) meetings. Finally, 7.7% (62 of 810) of published abstracts were presented at more than one meeting.

**Conclusions:**

Publication rates in this study were higher than previous reports in musculoskeletal oncology and comparable or better than recent reports for other orthopedic meetings. Comparisons across the AAOS, CTOS, and MSTS annual meetings highlight notable differences but suggest similarity overall in the quality of evidence presented with little overlap between meetings. Taken together, this study points to progress in the review processes used by the program committees, reaffirms the importance of critical appraisal when considering abstract findings, and supports the continued organization of multiple scientific meetings in musculoskeletal oncology.

## 1. Introduction

Scientific meetings provide an opportunity for scientists and clinicians to disseminate new research prior to publication in an environment that fosters innovation, collaboration, and debate. These forums are longstanding traditions and play an important role in advancing scientific discovery and patient care. New information is typically presented as an abstract, by podium or poster, following a peer-review selection process coordinated by the meeting program committee. Presentation is ideally followed by full-text publication in a peer-reviewed journal, which provides a more rigorous appraisal that meets the highest standards of scientific scrutiny. The quality of abstracts presented at a meeting can therefore be estimated by their publication rate.

The field of musculoskeletal oncology is comprised of many specialists, including orthopedic oncologists for whom the American Academy of Orthopaedic Surgeons (AAOS), Connective Tissue Oncology Society (CTOS), and Musculoskeletal Tumor Society (MSTS) annual meetings are frequently attended. Each of these meetings targets a slightly different audience but it is currently unclear how much overlap exists between meetings, and whether one meeting offers superior evidence over another. To our knowledge, only publication rates from the MSTS meeting have been examined previously. In a 2003 study, a review of select MSTS podium abstracts from 1991 to 1999 revealed a 41% publication rate [1]. The authors of this study strongly caution meeting attendees to “beware of the unpublished abstract”. They reason that the majority of abstracts presented may not survive rigorous peer review and, therefore, may not be scientifically valid. Overinterpretation of such abstracts, without a proper critical appraisal, can misdirect future research and clinical care. Examination of publication rates in other orthopedic subspecialties, notably trauma, demonstrate significant improvement over time [2, 3]. A more recent analysis of the MSTS meeting or related musculoskeletal oncology meetings has not been published. Such an analysis is desirable to set the stage for the judicious interpretation of abstracts, justify continued participation in multiple meetings, and evaluate progress over time. This study, therefore, asked the following: (1) what are the publication rates for oncology abstracts presented at the AAOS, CTOS, and MSTS annual meetings? and (2) what are the characteristics of publications following abstracts for each meeting?.

## 2. Methods

A complete collection of abstracts from the final programs of the AAOS (2013 to 2015), CTOS (2012 to 2014), and MSTS (2012 to 2014) annual meetings were retrieved from organizational websites and organized into a single database. The 2015 AAOS annual meeting, which occurred in March of 2015, was the most recent meeting included to allow for a four-year publication window at the time of review. All abstracts presented as a podium or poster at the oncology-focused CTOS and MSTS meetings during this time period were included in this study. For AAOS meetings, only podium abstracts presented during oncology sessions or poster abstracts classified under tumor were included. For each abstract, a PubMed (https://pubmed.ncbi.nlm.nih.gov) and Google Scholar (https://scholar.google.com) search was performed to determine if there was a full-text publication associated with the abstract. Searches included both abstract keywords and either the first or last author of the abstract.

An abstract was considered published if the direct comparison of the meeting abstract and the full-text publication contained substantial similarities in methodology and results within four years of abstract presentation, in accordance with previously described methods [1]. For example, if the presented abstract was altered in the published form, but still had the same focus, it was scored as a published abstract. Similarly, presented abstracts with data that was included as part of a larger publication were considered published. Overall, the criteria for publication were liberal. If no match was identified, the abstract was not considered published. To ensure the reproducibility of these methods, an independent search was first performed for ten abstracts by three authors (CDC, HAQ, and PJG) with complete agreement. One author (HAQ) then completed a search for each of the 1408 podium and poster abstracts included. Of these, 138 abstracts were identified as having uncertain publication status and underwent a second independent review by a more senior author (CDC) who made the final determination of publication status. All searches were conducted from April to November of 2019.

Abstract characteristics were collected, including the year of presentation, name of the meeting, presentation type (podium or poster), and country of origin. The program for the 2013 MSTS meeting did not include the country of origin. For abstracts meeting criteria to be considered published, publication characteristics were collected, including date of online publication, journal of publication, and the journal impact factor, as reported in the 2015 Journal of Citation Reports [4]. It was also noted if an abstract was published prior to the first day of the meeting or if a published abstract was presented at more than one meeting, the latter identified only if abstracts from multiple meetings resulted in the same publication. Time to publication was determined in months from the first day of the meeting to the date of online publication, excluding abstracts published before the meeting.

### 2.1. Statistical Analysis

Descriptive statistics provided include counts with proportions for categorical variables and median values with interquartile ranges for continuous variables. Categorical variables were analyzed using the chi-square test. Continuous variables were not normally distributed and were therefore analyzed using the Kruskal-Wallis test followed by Dunn's test for multiple comparisons. The cumulative incidence of publication over time was estimated using the reverse Kaplan-Meier method and compared across groups using the log-rank test. To visualize the overlap of published oncology abstracts presented at AAOS, CTOS, and MSTS meetings, an area-proportional Venn diagram was constructed using eulerAPE (University of Kent, Canterbury, UK) [5]. All other statistical and graphical analyses were performed using Prism 8 (GraphPad, La Jolla, CA, USA). All statistical testing was two-sided, with a *p* value less than 0.05 being considered significant.

## 3. Results

### 3.1. Publication Rates for AAOS, CTOS, and MSTS Oncology Abstracts

**Figure 1:**
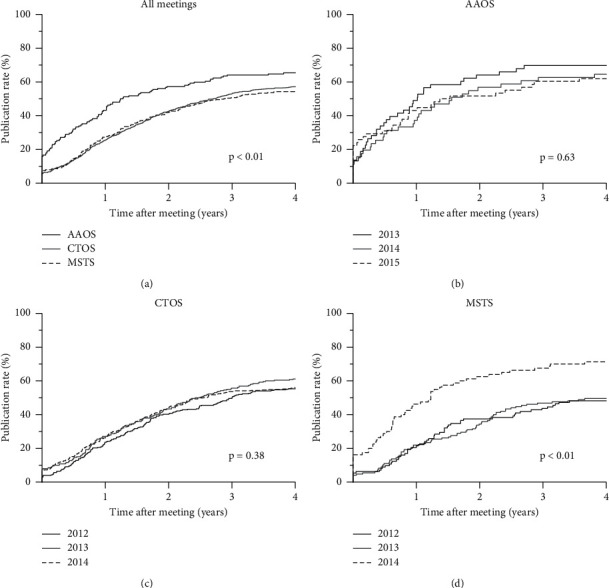
Publication rates over time for oncology abstracts presented at select AAOS, CTOS, and MSTS annual meetings are shown in (a). The publication rates over time are also shown for individual AAOS (b), CTOS (c), and MSTS (d) meetings. Time to publication was calculated as the interval from the first day of the meeting to online publication. Abstracts published prior to the first day of the meeting are depicted by the origin of each line on the *y*-axis. AAOS indicates the American Academy of Orthopaedic Surgeons; CTOS, Connective Tissue Oncology Society; MSTS, Musculoskeletal Tumor Society.

Abstract publication rates overall were higher for podium presentations (67%, 280 of 415) compared to poster presentations (53%, 530 of 993; *p* < 0.01). Differences in podium publication rates between the AAOS (69%, 66 of 96), CTOS (70%, 127 of 181), and MSTS (63%, 87 of 138) meetings were not statistically significant (*p*=0.39) (Table 1). Poster publication rates were statistically different and higher for AAOS meetings (AAOS: 61%, 40 of 60; CTOS: 54%, 394 of 728, MSTS: 48%, 96 of 199; *p*=0.04). When both abstract types were combined, differences between meetings did not meet statistical significance (AAOS: 65%, 106 of 162; CTOS: 57%, 521 of 909, MSTS: 54%, 183 of 337, *p*=0.06). Publication rates increased overall from year one after presentation (AAOS: 43%, 95% confidence interval (CI) 35% to 52%; CTOS: 26%, 21% to 30%, MSTS: 28%, 21% to 35%) to year two (AAOS: 57%, 95% CI 50% to 64%; CTOS: 43%, 39% to 46%, MSTS: 42%, 36% to 48%) to year three (AAOS: 64%, 95% CI 57% to 69%; CTOS: 52%, 49% to 55%, MSTS: 50%, 45% to 56%) in a time- and meeting-dependent manor (*p* < 0.01) (Figure 1(a)). Considering each meeting type separately, only the MSTS meeting demonstrated a significant change over time, with a higher overall publication rate for the smaller 2014 meeting (71%, 57 of 80) compared with the 2012 (48%, 54 of 112) and 2013 (50%, 72 of 145) meetings (*p* < 0.01) (Figures 1(b)–1(d)).

### 3.2. Characteristics of Publications Resulting from AAOS, CTOS, and MSTS Oncology Abstracts

**Figure 2:**
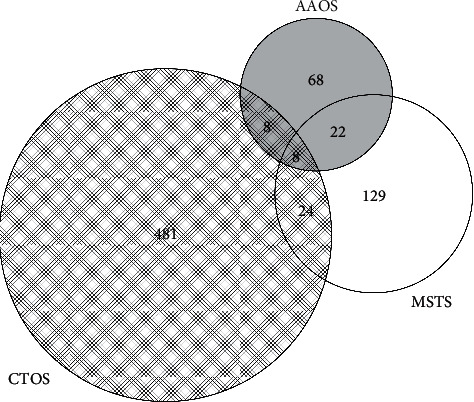
Overlap of published oncology abstracts presented at select AAOS, CTOS, and MSTS annual meetings. Depicted is an area-proportional Venn diagram, where the number of unique abstracts in each set is proportional to both the area of the circle and overlap. The number of unique abstracts in each set is also shown in the text. AAOS indicates the American Academy of Orthopaedic Surgeons; CTOS, Connective Tissue Oncology Society; MSTS, Musculoskeletal Tumor Society.

Abstracts from AAOS meetings were more often published prior to the first day of the meeting (AAOS: 24%, 25 of 106; CTOS: 10%, 52 of 521, MSTS: 14%, 25 of 183; *p* < 0.01). After excluding previously published abstracts, AAOS abstracts had the shortest time to publication (median: 10.8 months, interquartile range (IQR): 4.4 to 18.8 months), compared to those from CTOS (16.0 months, 8.4 to 25.9 months, *p* < 0.01) and MSTS (15 months, 7.9 to 25.0 months, *p* < 0.01) meetings. CTOS abstracts were published in higher impact journals (median: 3.7, IQR: 2.9 to 5.9), compared to those from AAOS (2.9, 1.9 to 3.2, *p* < 0.01) and MSTS (3.1, 2.3 to 3.1, *p* < 0.01) meetings. The most common journals of publication and countries of origin varied by meeting, as demonstrated in Tables 2 and 3, respectively. Finally, 7.7% (62 of 810) of published abstracts were presented at more than one meeting (1.0% [8 of 810] presented at AAOS, CTOS, and MSTS; 1.0% [8 of 810] presented at AAOS and CTOS only; 2.7% [22 of 810] presented at AAOS and MSTS only; and 3.0% [24 of 810] presented at CTOS and MSTS only) (Figure 2).

## 4. Discussion

The AAOS, CTOS, and MSTS annual meetings are important forums to advance research and patient care in musculoskeletal oncology. From 2012 to 2015, the overall publication rates for podium and poster presentations at these meetings were 67% and 57%, respectively. AAOS oncology abstracts were characterized by higher poster publication rates, publication prior to the meeting, and shorter time to publication. CTOS abstracts had longer time to publication and were published in higher impact journals. Only the MSTS meeting demonstrated a significant change in publication rates over time. Despite these notable differences, there was no statistically significant difference in the podium and overall publication rates between meetings or evidence of significant overlap in the information presented. Together, these findings suggest a continued role for multiple scientific meetings in musculoskeletal oncology.

There are important limitations to our study. First, the publication-search methodology, though modeled after similar studies, is inherently susceptible to missed or misidentified events that could have resulted in an underestimation or overestimation of publications rates and characteristics. Considerable efforts were made to mitigate this risk, including searching multiple abstract author names in two databases (PubMed and Google Scholar) by multiple authors of the current study, but the risk of missed or misidentified publications is not negligible. Second, abstracts resulting in publications that occurred more than four years after the meeting were not considered published abstracts, which allowed direct comparison of meetings over time. Though previous studies and our experience indicate that a publication resulting from an abstract presented over four years prior is rare, this may have resulted in an underestimation of publication rates. Third, though we reviewed 1408 abstracts over three years, a larger cohort with earlier meetings would have improved our assessment of trends over time. Despite these limitations, this study is strengthened by the direct comparison of three major meetings in musculoskeletal oncology, which has not been previously performed to our knowledge.

For all meetings included in this study, the publication rate was higher for podium presentations than poster presentations. This finding is consistent with prior reports on the AAOS, Orthopaedic Trauma Association (OTA), American Orthopaedic Society for Sports Medicine (AOSSM), and American Orthopaedic Foot and Ankle Society (AOFAS) annual meetings and suggests that podium presentations contain higher quality evidence that is more likely to survive the peer-review required for full-text publication [2, 6–8]. Comparing oncology abstracts from the three meetings reviewed here, the AAOS meetings had higher publication rates than CTOS or MSTS meetings, though this only reached statistical significance for poster presentations. Though not studied directly, this may be related to meeting size. The AAOS meetings and the highly published 2014 MSTS meeting had fewer presentations overall, which may have resulted from greater selectivity by program committees. Still, all publication rates reported in our study were higher than the 41% publication rate for MSTS podium abstracts from 1991 to 1999 [1]. This trend towards higher publication rates in recent years is reflected throughout orthopedics. Publication rates for national orthopedic meetings ranged from 34% to 67% in the 1990s, compared to 54% to 74% in recent reports, which is similar to our results [1–3, 8–16]. The OTA meeting is one well-studied example, with relatively high publication rates overall that increased from 67% (1994 to 1998) to 73% (2008 to 2012) for podium presentations [2, 3]. This increase corresponded to an increase in the level of evidence, as defined by commonly used evidence-based guidelines, presented over the same time period [17]. However, a similar study examining the level of evidence presented at MSTS meetings did not identify an improvement over time [18]. This suggests that the higher publication rates observed in our study are not related to improved levels of evidence. Alternatively, higher publication rates may reflect an improvement in the review process used by program committees, an increase in the number of journals available for publication, accessibility of open-access journals, more pressure to publish, or other unidentified factors.

There were some differences in the characteristics of publications resulting from AAOS, CTOS, and MSTS meetings. First, 24% of AAOS successfully published oncology abstracts were published prior to the meeting, and the remaining were published a median of 10.8 months thereafter, which was significantly faster than CTOS (16.0 months) and MSTS (15.0 months) abstracts. As noted previously, this finding may be related to the smaller number of abstracts presented at the AAOS meeting and greater selectivity that resulted in the presentation of more developed projects. It may also reflect the timing of the AAOS meeting, which accepts abstracts in the late summer before presentation in spring of the following year. By contrast, the CTOS and MSTS meetings accept abstracts in the late summer for presentation in the fall of the same year. The longer time interval between abstract acceptance and presentation for AAOS meetings allows more time for publication. Despite these differences, all meetings studied here had a shorter time to publication than the 21.8 months average previously reported for MSTS podium presentations from 1991 to 1999 [1]. Second, CTOS abstracts were published broadly and in significantly higher impact journals than AAOS or MSTS abstracts, most commonly in Annals of Surgical Oncology (5%), European Journal of Cancer (4%), and PloS One (4%). This finding likely reflects broader participation by medical oncologists, radiation oncologists, basic scientists, and other nonorthopedic surgeons at CTOS meetings, for whom higher impact journals are frequently targeted for publication. For both AAOS and MSTS, the most common journal of publication was Clinical Orthopaedics and Related Research, which accounted for 14% and 27% of publications, respectively. Clinical Orthopaedics and Related Research is the official publication of the MSTS and publishes proceedings from the MSTS meeting annually. Third, consistent with the organizational mission of CTOS to be international, 67% of CTOS abstracts originated from outside the United States. This is in contrast to the United States-based AAOS and MSTS, for which 49% and 35% of abstracts originated outside the United States, respectively. Finally, only 7.7% of published abstracts were presented at more than one meeting to suggest that the majority of the information presented at each meeting is unique. Therefore, each meeting serves a different audience, and together, they offer three unique programs to choose from, as attendees consider the best use of their time and resources.

## 5. Conclusions

In summary, publication rates in this study were higher than previous reports in musculoskeletal oncology and comparable or better than recent reports for other orthopedic meetings. These findings must be considered along with the recognition that many abstracts presented at national meetings are not subsequently published in peer-reviewed journals. Meeting attendees are therefore encouraged to think critically when digesting research from any source, particularly meeting abstracts, and ideally should weigh a body of evidence before altering research or clinical practices. Comparisons across the AAOS, CTOS, and MSTS annual meetings also highlight notable differences but reveal similarity overall in the quality of evidence presented with little overlap between meetings. These findings do not suggest that one meeting is superior or redundant. Taken together, this study points to progress in the review processes used by the program committees, reaffirms the importance of critical appraisal when considering abstract findings, and supports the continued organization of multiple scientific meetings in musculoskeletal oncology.

## Figures and Tables

**Table 1:** Publication rates and characteristics of oncology abstracts presented at AAOS, CTOS, and MSTS meetings.

	Meeting				Intergroup comparisons (*p* value)^		
	AAOS 2013–2015	CTOS 2012–2014	MSTS 2012–2014	Overall *p* value	AAOS/CTOS	AAOS/MSTS	CTOS/MSTS
Publication rate							
** **Podium	69% (66/96)	70% (127/181)	63% (87/138)	0.39	—		
** **Poster	61% (40/60)	54% (394/728)	48% (96/199)	0.04	—		
** **Both	65% (106/162)	57% (521/909)	54% (183/337)	0.06	—		
Published prior to meeting	24% (25/106)	10% (52/521)	14% (25/183)	<0.01	—		
Time to publication (months)+^*∗*^	10.8 (4.4–18.8)	16.0 (8.4–25.9)	15.0 (7.9–25.0)	<0.01	<0.01	0.01	>0.99
Journal impact factor (2015)^*∗*^	2.9 (1.9–3.2)	3.7 (2.9–5.9)	3.1 (2.3–3.1)	<0.01	<0.01	>0.99	<0.01

+Time to publication was calculated as the interval in days from the first day of meeting to online publication and excludes abstracts published prior to the meeting. ^*∗*^ Time to publication and journal impact factors are reported as median (interquartile range). ^ Intergroup comparisons were performed only for continuous variables after a Kruskal-Wallis test using Dunn's multiple comparisons test. AAOS indicates the American Academy of Orthopaedic Surgeons; CTOS, Connective Tissue Oncology Society; MSTS, Musculoskeletal Tumor Society.

**Table 2:** Top ten journals with most publications from select AAOS, CTOS, and MSTS annual meetings.

Meeting	Rank	Journal	Number of publications	% of total publications	Journal impact factor (2015)
AAOS					
	1	Clinical Orthopaedics and Related Research	15	14	3.13
	2	Journal of Surgical Oncology	8	8	3.15
	3	Journal of Bone and Joint Surgery	7	7	5.16
	4	Annals of Surgical Oncology	5	5	3.66
	5	PloS One	4	4	3.06
	6	European Journal of Surgical Oncology	3	3	2.94
	7	Anticancer Research	2	2	1.90
	7	BMC Cancer	2	2	3.27
	7	International Journal of Clinical Oncology	2	2	1.81
	7	Journal of Arthroplasty	2	2	2.51
	7	Journal of Orthopaedic Research	2	2	2.81
	7	Journal of Pediatric Orthopaedics	2	2	1.33
	7	Orthopaedics	2	2	1.13
	7	Sarcoma	2	2	2.26
	7	Spine	2	2	2.44
	7	The Bone and Joint Journal	2	2	2.66
	7	The Spine Journal	2	2	2.66
		Other	42	40	—

CTOS					
	1	Annals of Surgical Oncology	24	5	3.66
	2	European Journal of Cancer	21	4	6.16
	3	PloS One	19	4	3.06
	4	Cancer	17	3	5.65
	4	Journal of Surgical Oncology	17	3	3.15
	6	Clinical Cancer Research	16	3	8.74
	6	Oncotarget	16	3	5.01
	8	Annals of Oncology	13	3	9.27
	8	Clinical Orthopaedics and Related Research	13	3	3.13
	8	Journal of Clinical Oncology	13	3	20.98
	8	Pediatric Blood and Cancer	13	3	2.63
		Other	339	65	—

MSTS					
	1	Clinical Orthopaedics and Related Research	50	27	3.13
	2	Journal of Surgical Oncology	9	5	3.15
	2	Sarcoma	9	5	2.26
	4	Journal of Bone and Joint Surgery	8	4	5.16
	5	Annals of Surgical Oncology	4	2	3.66
	5	Bone and Joint Research	4	2	2.43
	5	Orthopedics	4	2	1.13
	5	PloS One	4	2	3.06
	9	Cancer Medicine	3	2	2.92
	9	Open Orthopaedics Journal	3	2	^*∗*^
	9	The Bone and Joint Journal	3	2	2.66
		Other	82	45	—

^*∗*^Impact factor not provided in the 2015 Journal of Citation Reports. AAOS, American Academy of Orthopaedic Surgeons; CTOS, Connective Tissue Oncology Society; MSTS, Musculoskeletal Tumor Society.

**Table 3:** Top ten countries with most abstracts from select AAOS, CTOS, and MSTS annual meetings.

Meeting	Rank	Country	Number of abstracts	% of total abstracts	Publication rate
AAOS					
	1	United States of America	82	51	70% (57/82)
	2	Japan	42	26	64% (27/42)
	3	Multiple countries	15	9	53% (8/15)
	4	Korea	7	4	71% (5/7)
	5	Italy	6	4	67% (4/6)
	6	United Kingdom	5	3	20% (1/5)
	7	Canada	1	1	0% (0/1)
	7	France	1	1	100% (1/1)
	7	Singapore	1	1	100% (1/1)
	7	Sweden	1	1	100% (1/1)
	7	Taiwan	1	1	100% (1/1)
		Other	0	0	—

CTOS					
	1	United States of America	303	33	59% (180/303)
	2	Multiple countries	165	18	68% (113/165)
	3	Japan	95	10	44% (42/95)
	4	France	46	5	63% (29/46)
	5	Italy	44	5	55% (24/44)
	6	Canada	42	5	62% (26/42)
	7	United Kingdom	38	4	26% (10/38)
	8	Germany	37	4	51% (19/37)
	9	Netherlands	24	3	54% (13/24)
	10	Korea	22	2	77% (17/22)
		Other	93	10	52% (48/93)

MSTS^*∗*^					
	1	United States of America	135	65	64% (87/135)
	2	Multiple countries	19	9	53% (10/19)
	3	United Kingdom	11	5	27% (3/11)
	4	Canada	10	5	90% (9/10)
	5	Argentina	7	3	71% (5/7)
	5	India	7	3	29% (2/7)
	7	Japan	4	2	100% (4/4)
	8	Chile	3	1	0% (0/3)
	9	Austria	2	1	50% (1/2)
	9	Sweden	2	1	50% (1/2)
	9	Switzerland	2	1	50% (1/2)
		Other	5	2	40% (2/5)

^*∗*^Countries were not available for the 2013 MSTS annual meeting. AAOS, American Academy of Orthopaedic Surgeons; CTOS, Connective Tissue Oncology Society; MSTS, Musculoskeletal Tumor Society.

## Data Availability

The datasets generated during and/or analyzed during the current study are available from the corresponding author on reasonable request.
